# 

**Published:** 1918-06-08

**Authors:** 


RATES OF SUBSCRIPTION: Twelve months, 6/6; Six months, 4/-; three months, 2/-, post free to any address in the United Kingdom. Abroad, Twelve months, 8/8; Six months, 5/-; Three months, 2/6, post free. THE HOSPITAL "Index" to Volume LXIII October 1917 to March 1918, is now ready, price 3½d. per copy, post free. Address The Manager, THE HOSPITAL, "The Hospital" Building, 28/29 Southampton Street, Strand, London, W.C. 2.

**URGENT NOTICE.**

To ensure a copy of THE HOSPITAL regularly, readers must place a definite order with their Newsagent TO DAY. After the middle of June, in consequence of the new Paper Restrictions Order, it will not be possible for Newsagents to stock copies for chance customers.

DO NOT DELAY. ORDER

"THE HOSPITAL" TO-DAY

and obtain your copy regularly EVERY THURSDAY.

### Medical Appointments.

Mr. Robert Arthur Young, M.D., F.R.C.P., has been elected a physician to the Middlesex Hospital, in succession to Dr. F. J. Wethered, who has resigned.

The vacancy of Assistant Medical Officer of Health to the Bethnal Green Borough Council, primarily for the purposes of the Maternity and Child Welfare Scheme, has been filled by the appointment of Miss Jeannie Rose Murray, M.B., B.Ch., B.A.O.R.U.I., 1906; D.P.H. Liverpool; Assistant M.O.H. Bradford Infant Clinic. Miss Murray has previously held the following appointments: Assistant House Surgeon, Victoria Children's Hospital, Hull; Resident Medical Officer at the Battersea Branch, Clapham Maternity Hospital and Dispensary for Women; Resident Medical Officer, Birkenhead Union Infirmary; Assistant School M.O. County Borough of Stoke-on-Trent; M.O. to the Child Welfare Centre, City of Manchester.

### THE COLLEGE OF NURSING (Limited by Guarantee).

### THE EDINBURGH CENTRE.

### Forty-Two Nurses Join at Meeting.

A meeting of all the members of the College of Nursing resident in Edinburgh and the district was held on Wednesday, May 29, at 19 Drumsheugh Gardens, the room having been kindly lent by the Committee of the Royal Scottish Nursing Institution. There was a very good attendance, and many nurses who could not be present wrote to give the meeting, whose purpose was to form a local branch of the College, their hearty support. Miss Gill, R.R.C., Lady Superintendent of the Royal Infirmary, who was in the chair, outlined the proposed scheme, and explained that the branch would be a centre for information regarding the College and all nursing matters, for social intercourse amongst its members, and for lectures on technical subjects. She hoped that the branch would also be of value as a recruiting centre for the College, and that the fact that nurses were thus banding themselves together might have a weighty influence on their position as women and as citizens. At the close of the speech the meeting resolved to form an Edinburgh branch of the College of Nursing, Ltd., with an Executive Committee of twenty-four members, twenty of whom, representing various nursing interests, were appointed forthwith. The following officers were also elected :—Miss Gill, President of the branch; Miss Graham, Matron of the Scottish Trained Nurses' Association, Chairman of the Executive Committee; and Miss Turnbull, R.R.C., Matron of the Deaconess Hospital, and Miss Cathcart, Joint Hon. Secretaries. The annual subscription was fixed at half-a-crown. Forty-two nurses joined at the meeting, and many have sent in their names since. Applications and inquiries should be addressed to Miss Cathcart, The Elms, Whitehouse Loan, Edinburgh.

### THE PLYMOUTH CENTRE.

### Mayor Declares College Commands Interest of Majority British Nursing Authorities.

A resolution approving of the College of Nursing and establishing a centre at Plymouth was unanimously adopted at a largely attended meeting held at the South Devon and East Cornwall Hospital, Plymouth, on Friday, May 31. The Mayor (Mr. J. P. Brown), who was in the chair, said that the establishment of the College had met with a certain amount of opposition, but having carefully weighed the evidence he had come to the conclusion that the College commanded the interest of the large majority of the nursing authorities of the country, and he therefore felt he was carrying out the wishes of the community in identifying himself with the organisation. By the establishment of the College the status of nurses would be secured, and their qualifications rendered indisputable. Through organisation, too, improvements in regard to pensions and other matters (including pay) should be ensured. Miss Cowlin, the Organising Secretary, told the meeting that already seven centres had been formed, and a voluntary Register of over 9,000 nurses had been compiled. The College movement stood for improved conditions for nurses throughout the country, and the attainment of State Registration, by which the public would be protected through the regulation of the standard of training. The College of Nursing was a big national scheme, worthy of whole-hearted support. Its aim was to uphold the highest ideals of the nursing profession. Captain Lindsay, R.A.M.C., and the Rev. J. P. Baker also spoke. A local committee, with power to add to its number, was appointed, including representatives of the following classes of hospitals and nursing institutions :—Military, Territorial, general, Poor-Law, infectious, special, and maternity hospitals; district, school, and private nursing; health visiting; convalescent homes and auxiliary hospitals. The first meeting of the committee is to take place at the Military Hospital, Devonport, on Friday, June 28, at 3.30, at the invitation of Miss Monck-Mason. At this meeting the executive officers will be elected, one of whom will act as a representative on the Council of the College of Nursing.

### TRURO CENTRE FORMED.

AT a small but enthusiastic meeting held on June 1 at the Royal Cornwall Infirmary, Truro, the Cornwall Centre of the College of Nursing was established. Miss Chaff, who was in the chair, said that Cornwall was made up of a scattered and often sparsely populated district. It consequently had its own peculiar problems to deal with, and the nurses in Cornwall felt this could only be done by the formation of their own centre. The centre, she emphasised, must inevitably be a small one, but would compensate for this by its enthusiasm. Miss Cowlin pointed out that although, for convenience sake, the headquarters of the College were in London, it was as much the Truro College of Nursing, or the Plymouth College, as the London College. It must, however, be realised that the privilege of self-government involved responsibility, and nurses must, without delay, fit themselves to take an intelligent part in the organisation of their own profession. Miss Chaff was appointed Local Representative, and Mrs. Woodruffe Hon. Secretary.

### St. George's Hospital.

A CONCERT is being arranged, through the permission of Mr. Mills, of the National Sunday League, to be held at the Palladium on Sunday, June 23, in aid of the funds of St. George's Hospital.

### Matrons' and Sisters' Appointments.

BOLTON INFIRMARY AND DISPENSARY.—Miss Marie Pape has been appointed sister. She was trained at the above institution, where she has since been charge nurse and holiday sister.

CHESTERFIELD JOINT HOSPITAL, CHESTERFIELD.—Miss Elsie Cooke has been appointed night sister. She was trained at the Monsall Fever Hospital, where she was later staff nurse.

COUNTY HOSPITAL, YORK.—Miss Alice Evans and Miss Hilda Anderson have been appointed sisters. Miss Evans was trained at the Royal Gwent Hospital, where she was later sister. She has also been sister at the Royal Surrey County Hospital, Guildford. Miss Anderson was trained at St. Bartholomew's Hospital, Rochester, where she had charge of a women's surgical ward, also holding the post of masseuse. She has also been night and home sister at the Royal Surrey County Hospital, Guildford.

CROYDON UNION INFIRMARY.—Miss Dorrit Katherine Fussell has been appointed night sister and acting assistant matron. She was trained at the Shoreditch Infirmary, and has been ward sister, night superintendent, theatre and home sister at Kensington Infirmary.

INFECTIOUS HOSPITAL, TAMWORTH.—Miss Bertha Coury has been appointed matron. She was trained at Addenbrooke's Hospital, Cambridge, has held posts under local authorities as school nurse, health visitor and school inspector, and has been later matron of the Military Hospital, Whitley, Chester.

INFIRMARY AND DISPENSARY, ROCHDALE.—Miss Elsie Ball has been appointed assistant matron. She was trained at the Hope Hospital, Pendleton, where she was later ward and theatre sister, and where she gained the Ingleby gold medal. She has also been sister of a male ward at Rochdale Infirmary.

KING'S COLLEGE HOSPITAL, LONDON.—Miss Florence Hancock has been appointed sister-housekeeper. She was trained at King's College Hospital, where she was later night and ward sister. She has also been a Queen's nurse and a sister under the Territorial Force Nursing Service at the 4th London General Hospital.

MOSTYN CONVALESCENT HOME, HOLYWELL.—Miss E. R. Smythe has been appointed sister. She was trained at the South Manchester Hospitals.

ROYAL INFIRMARY, BRADFORD.—Miss Flora Hele has been appointed theatre sister. She was trained at the Middlesex Hospital and the London Fever Hospital, and has been theatre sister at the Middlesex Military Hospital.

ROYAL INFIRMARY, SUNDERLAND.—Miss L. Wilkinson has been appointed night superintendent. She was trained at Lincoln County Hospital, and has since been theatre and ward sister at York County Hospital.

ROYAL LIVERPOOL COUNTRY HOSPITAL FOR CHILDREN, HESWALL, CHESHIRE.—Miss Maud Lightbown has been appointed sister. She was trained at the Royal Infirmary, Oldham, and has been theatre, night, and massage sister at the General Infirmary, Rochdale.

**NOTE.—Particulars of Appointments for insertion in this column will be welcomed.**

### Lectures on Venereal Diseases.

THE demonstrators at the course of special demonstrations and discussions on venereal disease at the Laboratories of the Royal Institute of Public Health, 37 Russell Square, on Wednesdays, June 12 to July 3, at 2.30 P.M., are :—Mr. Aubrey H. Drew, Mr. A. W. Stewart, D.Sc., Mr. Jas. H. Sequeira, M.D., Mr. J. E. Barnard. At St. Paul's Hospital for the Skin, 13A Red Lion Square, Holborn, W.C. 1, a course of free lectures to nurses is being given by Mr. Leonard Myer, F.R.C.S., on Tuesdays, June 11, 18, 25, July 2 and 9, at 5 P.M.

